# At the Frontiers of Neurorehabilitation, Series II: Advancing Neurorehabilitation Through Patient-Centered Undogmatic Innovative Approaches

**DOI:** 10.3390/brainsci14121222

**Published:** 2024-12-02

**Authors:** Hélène Viruega, Manuel Gaviria

**Affiliations:** 1 Institut Equiphoria, Combo Besso Rouges Parets, 48500 La Canourgue, France; helene.viruega@equiphoria.com; 2 Clinical Neurosciences, Alliance Equiphoria, 4 Res du Sabot, 48500 La Canourgue, France

**At the Frontiers of Neurorehabilitation: Series II**

Neurorehabilitation is undergoing a transformative shift, with scientific and technological advances creating new pathways for treating and supporting recovery in individuals with neurological disabilities. This field now encompasses a wide range of conditions, from traumatic and vascular brain injuries to spinal cord injuries, degenerative disorders like Parkinson’s disease and multiple sclerosis, and chronic issues such as neuropathic pain and post-traumatic stress disorder. The expansion of neurorehabilitation reflects a multidisciplinary approach driven by an evolving understanding of neuroplasticity—the brain’s remarkable capacity to reorganize and adapt [1,2,3]. Today’s therapeutic strategies aim not only to ease symptoms but also to promote brain remodeling and rewiring its functions, enhancing the pursuit of greater autonomy for each individual.

Historically, neurorehabilitation has focused on strengthening residual capacities and compensating for lost functions relying on the intrinsic brain capacities. However, recent years have seen a boom in new technologies that are redefining therapeutic limits. Innovations in neurostimulation, robotics, virtual and augmented reality, brain-computer interfaces and sensory feedback systems, for example, are providing patients with tools and strategies that can accelerate their recovery enabling new forms of adjustment that complement the more traditional results linked to the alleviation of functional deficits and strengthen their commitment to their rehabilitation journey [4,5]. Each of these innovations represents a way of treating specific neurological deficits and improving patients’ autonomy and quality of life.

Neurorehabilitation researchers and clinicians are progressively aware of the critical role psychological and mental functioning play in physical recovery. For instance, emotional dysregulation, chronic pain, cognitive impairments and post-traumatic stress disorder can create significant obstacles to rehabilitation becoming invisible barriers. Addressing these aspects must be an integral component of therapeutic design. Also, it is agreed that a lack of environmental enrichment negatively impacts functional recovery and might be a critical factor in time-dependent neurological decline [6,7].

At the heart of these advances a new vision arises: rehabilitation should be stimulating, adaptive and patient-centered [8]. It is increasingly recognized that individualized therapies, tailored to a patient’s specific needs and interests, are more effective than one-size-fits-all approaches [9]. The studies presented in this special issue “At the frontiers of neurorehabilitation: Series II” reflect this mindset, presenting cutting-edge approaches that combine conventional therapeutic methods with technology and creative engagement. In bringing together this selection of papers, we aim to offer insights into the evolving landscape of neurorehabilitation and promote continued research and innovation.

The articles featured present a wide array of approaches, beginning with Salera and colleagues’ study on the ***Michelangelo Effect in Cognitive Rehabilitation: Using Art in a Digital Visuospatial Memory Task***. This research demonstrates how artistic stimuli can improve visuospatial memory performance, reducing perceived effort in both healthy individuals and patients, which has significant implications for designing cognitive rehabilitation programs that enhance motivation and engagement. This theme of engaging patients through novel means is echoed in ***Is Virtual Reality Orientation Therapy Useful to Optimize Cognitive and Behavioral Functioning Following Severe Acquired Brain Injury? An Exploratory Study***, where De Luca and colleagues. extend this exploration into the digital realm with their work on Virtual Reality Orientation Therapy for Severe Acquired Brain Injury (SABI). They explore how reality orientation therapy—using VR to reinforce orientation through time, place, and person-specific stimuli—supports cognitive and behavioral improvements in patients with SABI. This investigation into virtual therapy tools continues with ***Treadmill Training Plus Semi-Immersive Virtual Reality in Parkinson’s Disease: Results from a Pilot Study***, a study led by Pullia and colleagues. By combining treadmill exercises with virtual reality, the authors show promising results in reducing postural instability and improving gait, underscoring VR’s potential in addressing the complex needs of Parkinson’s patients. ***Efficacy of a Soft Robotic Exoskeleton to Improve Lower Limb Motor Function in Children with Spastic Cerebral Palsy: A Single-Blinded Randomized Controlled Trial***, by Zhichong Hui and colleagues, highlights the therapeutic potential of lightweight, portable soft robotic exoskeletons. Their findings show that long-term, exoskeleton-assisted walking can effectively improve motor functions in children with spastic cerebral palsy, a breakthrough for pediatric rehabilitation. The research by Akaiwa and colleagues ***Does 20 Hz Transcranial Alternating Current Stimulation over the Human Primary Motor Cortex Modulate Beta Rebound Following Voluntary Movement?*** examines the effects of transcranial alternating current stimulation on beta rebound—a critical indicator of motor function recovery—suggesting therapeutic applications for enhancing motor recovery after neurological impairments.

Focusing on innovative concepts in motor rehabilitation, Raglio and co-authors introduce ***Movement Sonification Techniques to Improve Balance in Parkinson’s Disease: A Pilot Randomized Controlled Trial***. In this pilot study, musical auditory cues are shown to help Parkinson’s patients improve balance, suggesting that incorporating melodic-harmonic elements into neuromotor rehabilitation may further assist with balance and coordination. La Rosa and collaborators address one of the core objectives in neurorehabilitation in their paper ***Gait Recovery in Spinal Cord Injury: A Systematic Review with Meta-Analysis Involving New Rehabilitative Technologies***. This comprehensive analysis reviews how robotic technologies are advancing gait recovery in patients with spinal cord injuries, bringing hope for more accessible, effective rehabilitation tools to support functional independence.

In another approach to neurorehabilitation through mental practice, Fujiwara and colleagues’ study on ***Differences in Cortical Area Activity and Motor Imagery Vividness during Evaluation of Motor Imagery Tasks in Right and Left Hemiplegics*** sheds light on how motor imagery exercises can activate cortical areas differently depending on whether patients suffer from right or left hemiplegia. These insights can help refine therapeutic strategies that use motor imagery to support mental and physical recovery.

A different perspective on motor rehabilitation is explored in ***Exploring Methodological Issues in Mental Practice for Upper-Extremity Function Following Stroke-Related Paralysis: A Scoping Review***, by Akira Nakashima and colleagues. Their scoping review systematically maps out mental practice applications for stroke-induced paralysis, offering insights on optimal timing and technique to maximize recovery.

Kumar’s work, ***Adults with Cerebral Palsy: Navigating the Complexities of Aging***, highlights the unique challenges faced by adults with cerebral palsy, such as managing long-term motor deficits and finding accessible, specialized rehabilitation services. This narrative review brings attention to the gap in resources for aging CP patients, suggesting that a holistic and long-term care approach is essential for preventing functional decline and improving quality of life.

Lastly, in cognitive neuroscience, Lee and colleagues present a unique investigation in ***Unveiling Neurocognitive Disparities in Encoding and Retrieval between Paper and Digital Tablet-Based Learning***. This study delves into how the brain processes information differently when learning from paper versus tablets, highlighting implications for cognitive and educational practices, especially for digital learning in neurorehabilitation settings.

The works in this Special Issue collectively underscore the critical role of personalization in neurorehabilitation. Tailoring rehabilitation approaches to patients’ somatic, mental and emotional needs through cutting-edge original therapies yields promising outcomes and paves the way for integrating adaptive concepts, devices and technologies. From using wearable soft robotics to introducing virtual reality and brain stimulation through different sources, each study reaffirms that neurorehabilitation must remain creative, dynamic, responsive, and patient-centered. Leadership and insights into CNS plasticity have guided this exploration, advancing the conversation on adaptive neuroplasticity, the potential for innovation, and a renewed commitment to improving the autonomy and quality of life for those affected by neurological conditions.

We invite readers to explore these contributions, which aim not only to deepen scientific understanding but also to inspire further research and clinical practices in neurorehabilitation. Through ongoing research and collaboration, we look forward to a future where patients benefit from accessible, effective, and innovative rehabilitation solutions that empower them to regain autonomy and enhance their self-confidence. These new approaches give the opportunity to heavily disabled patients to experience soft and non-intrusive therapies allowing them to be closer to a “normal life”.
